# Spatial Effects and GWA Mapping of Root Colonization Assessed in the Interaction Between the Rice Diversity Panel 1 and an Arbuscular Mycorrhizal Fungus

**DOI:** 10.3389/fpls.2019.00633

**Published:** 2019-05-17

**Authors:** Hazel Davidson, Roshi Shrestha, Thomas Cornulier, Alex Douglas, Tony Travis, David Johnson, Adam H. Price

**Affiliations:** Institute of Biological and Environmental Sciences, University of Aberdeen, Aberdeen, United Kingdom

**Keywords:** *Oryza sativa*, *Rhizophagus irregularis*, *Glomus intraradices*, common mycorrhizal network, GWA mapping, QTL

## Abstract

If water saving methods of rice management are to be adopted, the interaction between rice plants and arbuscular mycorrhizal (AM) fungi will grow in agronomic significance. As yet there are very few studies on the interaction between rice and AM fungi and none on host genetics. A subset 334 cultivars from the Rice Diversity Panel 1 were grown in 250 L boxes filled with phosphorus (P) deficient aerobic soil without addition, with added rock phosphate and with rock phosphate and the AM fungus *Rhizophagus irregularis*. Statistical analysis of position of plants revealed a positive effect of their neighbors on their dry weight which was stronger in the presence of rock phosphate and even stronger with rock phosphate and AM fungi. A weak but significant difference in the response of cultivars to AM fungus treatment in terms of shoot dry weight (SDW) was revealed. Neighbor hyphal colonization was positively related to a plant’s hyphal colonization, providing insights into the way a network of AM fungi interact with multiple hosts. Hyphal colonization ranged from 21 to 89%, and 42% of the variation was explained by rice genotype. Colonization was slightly lower in *aus* cultivars than other rice subgroups and high in cultivars from the Philippines. Genome wide association (GWA) mapping for hyphal colonization revealed 23 putative quantitative trait loci (QTLs) indicating there is an opportunity to investigate the impact of allelic variation in rice on AM fungal colonization. Using published transcriptomics data for AM response in rice, some promising candidate genes are revealed under these QTLs being a calcium/calmodulin serine/threonine protein kinase at 4.9 Mbp on chromosome 1, two ammonium transporters genes at 24.6 Mbp on chromosome 2 and a cluster of subtilisin genes at 1.2 Mbp on chromosome 4. Future studies should concentrate on the biological significance of genetic variation in rice for AM colonization.

## Introduction

The symbiotic interaction between land plants and mycorrhizal fungi is ancient and is thought to be driven by the provision of nutrients (particularly P and N) to the plant in exchange for carbon (Smith and Read, 2008) in the form of sugar and lipids (Keymer and Gutjahr, 2018). Most grasses, including major crop plants, form symbioses with arbuscular mycorrhizal (AM) fungi that can affect agricultural plant productivity (Van Der Heijden et al., 2015), but research on the interaction between the grass crop rice and AM fungi has been very limited. This is probably because in flooded soil, in which most rice is grown, this association is traditionally considered unimportant. It has, for example, been shown that in flooded conditions AM fungi are very scarce on rice (Ilag et al., 1987) while it has been demonstrated that flooding of aerobically-grown rice roots decreases AM fungal colonization within 7 days- although it does not eliminate AM fungi completely (Vallino et al., 2014). Fieldwork has begun to provide evidence for an impact of AM fungi on rice productivity in upland (aerobic) soils (Maiti et al., 2011) but not for flooded rice. Yet as water availability and sustainability of crop production grow as drivers for crop management decisions [see for example page 8–9 in Bouman et al. (2007)], it can be predicted that increasing amounts of the world’s rice production will adopt one of a number of water saving techniques including aerobic rice, alternate wetting and drying or the system of rice intensification (SRI) (Bouman et al., 2007, p. 19–28). Under these growing conditions, rice-AM fungi interactions are more likely to be important. Indeed it has recently been shown that AM fungi colonization and AM fungal species richness were substantially higher under SRI (which includes a more aerobic soil condition) than conventional flooding (Watanarojanaporn et al., 2013). So it is timely to investigate the interaction more closely in order to consider the possibility that future rice breeding might need to incorporate the knowledge gained.

Molecular characterization of the AM fungi-rice interactions has provided insight into the biology of the interaction (Güimil et al., 2005; Campos-Soriano et al., 2012; Gutjahr et al., 2015). However, there has been almost no studies investigating if there are differences between rice cultivars in their interaction with AM fungi and no studies on the host genetics of the interaction. In a comparison of six upland cultivars from China, one was found to have much lower colonization when infected by either of two AM fungi (Gao et al., 2007), while Li et al. (2016) found differences in colonization of six cultivars with *Rhizophagus intraradices*. In the field in Italy no differences in colonization between 12 cultivars was detected (Vallino et al., 2009). Suzuki et al. (2015) assessed the growth response of 64 cultivars of rice to *Funneliformis mosseae* and found wide differences (from −4 to +119%). The authors assessed colonization in 12 cultivars (names not specified) and although this did differ between cultivar it did not related to the degree of growth response. We are not aware of any other studies that assessed differences in colonization between rice cultivars.

While there have been no studies on genetic mapping AM fungal interaction in rice, there have been on other crops. Leiser et al. (2016) found very little evidence of QTLs for AM colonization in sorghum probably because of weak repeatability between replicates. In contrast, Lehnert et al. (2017) examining wheat found strong genetic variation but still rather weak evidence of quantitative trait loci (QTLs) suggesting AM colonization is controlled by small effect genes, meaning QTLs and potential candidate genes reported are speculative. Finally, De Vita et al. (2018) screened 108 durum wheat cultivars for colonization by *R. irregularis* and *F. mosseae*, detecting substantial variation and seven putative QTLs by genome wide association (GWA) mapping.

In this study, the well characterized global collection of rice cultivars, the Rice Diversity Panel 1 (Zhao et al., 2011) was screened for growth and colonization in response to inoculation with the ‘model’ AM fungus *R. irregularis* (formerly known as *Glomus intraradices*). This species has been found colonizing aerobically-grown rice roots in India (Bhattacharjee and Sharma, 2011) and dominating them in Italy (Vallino et al., 2009), and was the first AM fungus sequenced (Tisserant et al., 2013; Van Der Heijden et al., 2015).

The main aim of this study was to test the hypothesis that there is genetic variation within rice for the colonization and the impact of AM fungus *R. irregularis*, and that these are correlated. Further, we test the hypothesis that genetic variation for AM fungal colonization can be genetic mapped. This was done using 334 accessions of the Rice Diversity Panel 1, allowing GWA mapping since there are 5.2 million SNP markers available for this population (Wang et al., 2018). To provide phenotype data that are relevant, discriminating and achievable required that a time-efficient screening system be developed. The design of the boxes used, specifically the competitive nature of the arrangement of plants, allowed three further hypotheses to be tested; (1) that spatial relationships between the rice plants affect the growth of the rice plant, (2) that the degree of neighbor interactions is different if an AM fungus is present, and (3) that AM fungal colonization rates are affected by the size and AM fungal colonization of neighboring plants.

## Materials and Methods

### Rice Genotypes

A random set of 334 accessions of the Rice Diversity Panel 1 (RDP1) (Zhao et al., 2011) was used for this study (see Supplementary Table S1). RDP1 represents 372 cultivars which come from the five subpopulations of rice (indica, aus, tropical japonica, temperate japonica, and aromatic). The cultivars have been genotyped using the High-Density Rice Array (HDRA) of 700,00 SNPs, with ∼1 informative SNP per kb (McCouch et al., 2016) and this data base has recently been extended to 5.2 million SNPs by imputation (Wang et al., 2018). In addition to the RDP1, cultivars Azucena and Bala which have been continuously grown in Aberdeen from seed originally obtained from the International Rice Research Institute were used as local check cultivars. These are distinguished from the Azucena and Bala in the RDP1 population by referring to them as “Own Azucena” and “Own Bala.”

### AM Inoculum

*Rhizophagus irregularis* inoculum was purchased from INVAM, United States and amplified in maize by growing three plants per pot in four pots of 2.5 L capacity filled with fine sand, each pot receiving 60 g of inoculum. These were fed weekly with zero P Yoshida’s nutrient solution (Yoshida et al., 1976) and on two occasions 1/4 P was included in the nutrient solution. After 3 months growth, when 100% hyphal colonization was confirmed in subsampled roots, the maize plants were dried by withholding water, the roots cut into fine pieces and the roots and sand used as inoculum.

### Soil

Throughout, the soil used was a mixture of 1 part subsoil from Insch Field Farm [previously described in MacMillan et al., 2006 (available P 12.24 μg g^–1^, total P 814 μg g^–1^)] and 3 parts fine sand (Sibelco UK; available P 1.95 μg g^–1^, total P 12.2 μg g^–1^). This was chosen as previous research had shown it suitable for experiments on P response (Al-Ogaidi, 2013).

### Greenhouse Conditions

All experiments were conducted in the Cruickshank Greenhouse of the University of Aberdeen where the temperature is set to 25°C and plants received ambient light plus 12 h a day of supplementary light of approximately 150 μM m^–2^ s^–1^ PAR.

### AM Colonization Measurement

The proportion of roots containing hyphae, arbuscules or vesicles of the AM fungus was assessed using the magnified intersections method of McGonigle et al. (1990) and slight modifications of the staining method of Vierheilig et al. (1998). Roots were washed to be free of soil and stored in 60% ethanol. Approximately 8–12 fine root sections of 3–4 cm in length were selected at random from the central portion of the root. After washing out the ethanol, they were bathed in 10 ml of 10% KOH at 90°C for 10 min. After clearing, samples were rinsed in cold water and acidified by dipping in 1% HCl for 30 s and then transferred to test tubes containing 8 ml of 1% ink (Parker’s Quink Black) in 1% HCl at 85°C for 4 min. This was followed by degassing under vacuum (while still in the stain) for 2 min. Samples were then washed in water to remove excess stain. Finally, they were transferred into sample storage jars containing lactic acid and glycerol (1:1:14 glycerol:water:lactic acid) de-stain for at least 24 h. These were mounted on microscope slides in glycerol. The proportion of approximately 100 root-graticule intersections observed for each plant containing a visible fungal hyphae, or an arbuscle or a vesicle was recorded. Hyphal colonization was calculated as the sum of all three of those since an arbuscle and a vesicle must be accompanied by a hyphae.

### Optimizing the Screening Method

Four preliminary experiments were conducted in order to optimize the screening methodology.

#### Dose Response

A dose experiment was conducted in the greenhouse using 1 L pots where 200 ml of soil/sand mix was placed at the bottom, 500 ml was placed in the middle and 100 ml on top. Different concentrations of inoculum were incorporated into the middle portion to give seven treatments being of 0, 1, 2, 5, 10, and 20% with an addition 10% autoclaved inoculum. Four replicates were used. Surface sterilized seeds of Azucena were placed in each pot and grown for 8 weeks with 200 ml of Yoshida’s nutrient minus P being given twice a week. SDW and colonization were assessed. This revealed no impact of the autoclaved inoculum on plant growth, AM fungi substantially reduced growth, 1% inoculum had a smaller effect, while the other treatments were identical. Hyphal colonization were not different between 2, 5, and 20% inoculum. No more than 2% was therefore considered an appropriate rate of inoculation. Importantly, no hyphae were observed in the 0 and 10% autoclaved treatments (in 12–14 roots of 4 replicate plants of both treatments) indicating the subsoil used is not a source of AM inoculum.

#### Time Course

**FIGURE 1:**
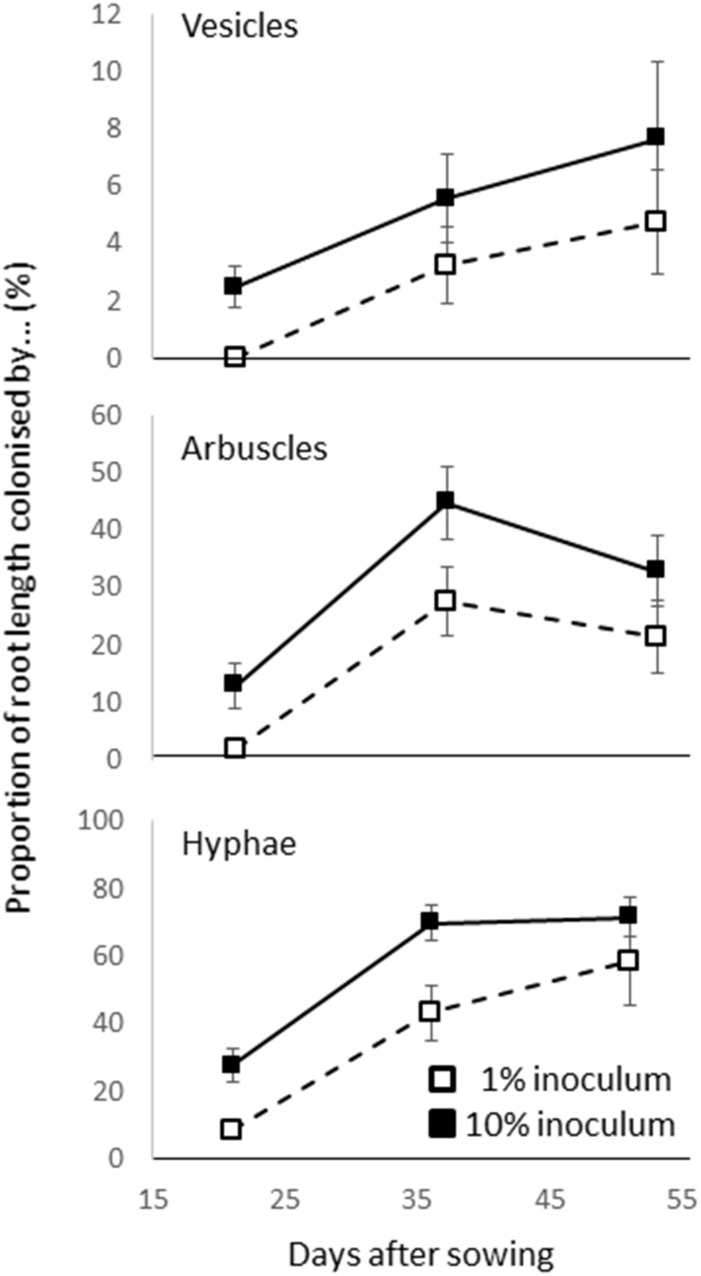
Arbuscular mycorrhizal (AM) colonization of roots in preliminary time course experiment in which cultivar Azucena was inoculated with 1 and 10% inoculum and the presence of vesicles, arbuscles, and hyphae were assessed on three occasions. Bar is standard error (*n* = 4).

A time course was conducted in which 1 and 10% inoculation (using the same pots, soil, filling methods, rice genotype and growth conditions as described above) were harvested at 21, 36, and 51 days. Arbuscles, vesicles, and hyphal colonization was assessed (Figure 1). The colonization rate increased with time (from 19–65%) but while 1% inoculum consistently showed a lower colonization rate than 10% inoculum, there was no interaction between dose and time (based on two way ANOVA *P* > 0.05). It was considered that a dose of 1% or more and a 4 weeks growth period should be suitable for studying the genetics of AM fungus-rice interactions.

#### AM Fungi and Rock Phosphate

A third preliminary experiment was conducted to examine the interaction between AM fungus and rock phosphate using pots, soils, soil filling, rice genotype and plant growth conditions as above. Treatments with 0, 50, 100, 200, and 300 mg of rock phosphate per pot were applied with and without AM fungi at 2% with four replicates. At 4 weeks, SDW and leaf P uptake were assessed while the AM colonization was assessed on the AM fungus treatment. AM treatment reduced SDW by 25% but rock phosphate did not affect it. AM fungus reduced leaf P concentration (from 1.47 to 1.08 mg g^–1^: *P* < 0.001) while rock phosphate increased it (from 1.08 to 1.46 mg g^–1^: *P* = 0.022) and there was no interaction. Rock phosphate increased hyphal colonization in a linear fashion (0 = 27%, 50 = 36%, 100 = 42%, 200 = 49%, and 300 = 61%; *P* = 0.035). This experiment suggested adding rock phosphate did not affect growth of these plants at this young age but did increase AM colonization.

#### Genetic Variation in Two Cultivars

A final preliminary experiment determined if there were genetic differences in colonization rates by testing cultivars Azucena and Bala [parents of the mapping population described in Price et al. (2000)]. Zero and 300 mg rock phosphate and zero and 2% AM fungus inoculation were used in a fully factorial design with four replicates. Other conditions were as above. Root and shoot fresh weight (not dry weight) were recorded. The AM fungus reduced the root weight of both cultivars (*P* = 0.010) but it did not affect shoot weight while rock phosphate increased shoot weight (*P* = 0.013) but did not affect root weight. There was a highly significant genotype by rock phosphate by AM fungus interaction for % root mass (root fresh weight/total fresh weight × 100) (*P* = 0.004) which was high in both cultivars in the absence of rock P (65–67%) but was lowest for Azucena with rock phosphate and AM fungus (61%) and lowest in Bala with rock phosphate but without AM fungus (58%). There was a highly significant difference in hyphal colonization rate between Bala and Azucena (*P* < 0.001) (Azucena 55.1%, Bala 30.4%), and a significant interaction between rock phosphate and cultivar (*P* = 0.010) where colonization was halved in Bala by rock phosphate (from 40.7 to 20.1%) but Azucena was not affected. It was concluded that treatment with rock phosphate may increase the ability to discriminate between cultivars.

### Screening the Rice Diversity Panel 1

**FIGURE 2:**
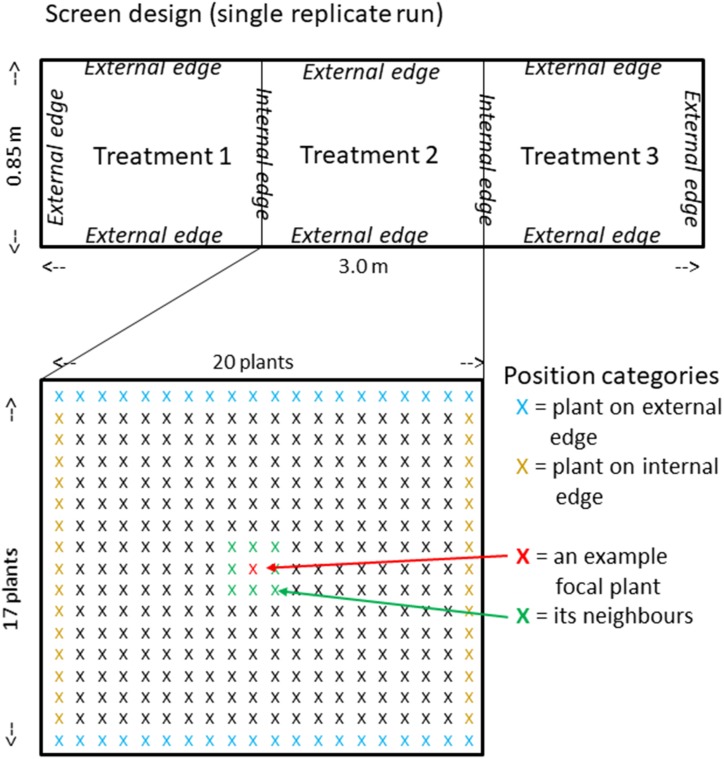
RDP1 screen design. Above, plan of the three treatments (randomly allocated) of each replicate run showing internal and external edges. Below, expanded plan of single central treatment showing individual plants (marked with X) highlighting plants on external and internal edges, plus a graphic explanation of the meaning of neighbors in relation to a focal plant.

Boxes were built from plywood 3 m long, 0.85 m wide and approximately 0.35 m deep and lined with plastic sheet. They were then divided into three 1 m long sections orientated north to south using stiff plastic and each section filled with subsoil/sand mix according to three treatments to a depth of 0.3 m (approximately 250 L of subsoil/sand in each) (Figure 2). A control treatment had no additions, a rock phosphate treatment (RP) had 100 mg per plant placed in a band in the top 10 cm and a rock phosphate and AM fungus treatment (RP + AM) had 100 mg per plant rock phosphate and 1% AM fungal inoculum placed in a band in the top 10 cm. Two surface-sterilized seeds of 334 cultivars of the Rice Diversity Panel 1 were sown completely randomized in a grid pattern of 17 × 20 with a spacing of 5 cm between plants. The same randomization was used for each of the three treatments within a replicate run. After germination, plants were thinned to one. Plants were watered daily while every other day they received Yoshida’s nutrient solution without P so that they received approximately 0.125 L each (equivalent to 5 mg of N which would produce a plant of approximately 0.25 g shoot dry weight (SDW) if not restricted by P). After 4 weeks growth, shoots were harvested for dry weight measurement while for the AM treatment alone the roots were harvested for colonization assessment. The experiment was repeated in four replicate runs starting on 15th May, 29th May, 9th July, and 23rd July 2013 each with a different randomization of the 334 genotypes. The order of the treatments was also randomized between runs and consisted of, from south to north run 1 Control:RP + AM:RP; run 2 AM + RP:RP:Control; run 3 RP + AM:RP:Control; run 4 RP:Control:RP + AM. Note AM hyphal colonization were assessed on 4–6 plants from the control and RP treatment in every run. On one occasion, 12% colonization was found, but the vast majority of samples contained no evidence of colonization.

### Spatial Statistics

Spatial and genetic variation in SDW was modeled using generalized additive mixed models, assuming a Gaussian error after log transformation, in order to stabilize the variance. Hyphal colonization (%) was modeled using generalized additive mixed models, assuming a Gaussian error. The models included a combination of fixed, smooth and random effects listed in Table 1, as well as second-order interactions between the fixed effects. The models were fitted by REML with the gamm and gamm4 functions of the R packages ‘mgcv’ and ‘gamm4,’ respectively (Wood et al., 2013). For the SDW analysis, the ‘Variety’ random intercept was nested in ‘Subgroup,’ and a genotype by treatment interaction was evaluated by fitting the corresponding random effect (see Table 1). We tested if separate spatial trends (smoothing splines) were required for each block, box or run, or if all 4 runs could be suitably described by a common spatial trend, using the Bayesian Information Criterion as an indication of most parsimonious model. For the hyphal colonization analysis, we used a ‘Variety’ random intercept nested in ‘Subgroup.’ Spatial dependence of hyphal colonization of the focal plant on the colonization of its immediate neighbors was modeled using a Markov random field (MRF) smoother within each block. For both analyses, fixed effects were selected or discarded based on the significance of the p-values of their coefficients (Null hypothesis: coefficient value is zero).

**TABLE 1:** Predictors used in the spatial statistical models.

Variable name	Type	Fixed/random	Levels	Definition
Run*^§^	Categorical	Fixed	1, 2, 3, 4	Time block over which experiment was run
Treatment*	Categorical	Fixed	RP, AM + RP, Control	Experimental treatment
Treatment by Variety*	Categorical	Random (by Variety)	RP, AM + RP, Control	Genotype by treatment interaction
Edge*^§^	Categorical	Fixed	0 (Away from edge), 1 (Along edge)	Location of plant with respect to box edge
Exterior*^§^	Categorical	Fixed	0 (Away from edge), 1 (Along external edge of box)	Is plant on an external box edge (as opposed to internal box edge separating treatment blocks)?
Neigh.SDW*^§^	Numeric	Fixed		Mean SDW of 1st-order neighbors
X, Y*	Numeric	Bivariate smoothing spline		Describes smooth spatial trends in response variable
MRF^§^	Categorical	Markov random field	One per observation	Describes the dependence of focal plant hyphal colonization (HC) on neighbors HC (spatial autocorrelation)
Run*	Categorical	Indicator for the smoothing spline	1, 2, 3, 4	When used, a separate smooth spatial trend was estimated for each run
Box*	Categorical	Indicator for the smoothing spline	1, 2	When used, one smooth spatial trend was estimated for each breeding box (holding 3 treatments each)
Block*^§^	Categorical	Indicator for the smoothing spline	12 levels (3 treatments × 4 runs)	When used, one smooth spatial trend was estimated for each treatment block within a breeding box
Subgroup*^§^	Categorical	Random	7 levels: (*n* = 132), “ADMIX” (*n* = 417), “AROMATIC” (*n* = 144), “AUS” (*n* = 648), “IND” (*n* = 759), “TEJ” (*n* = 984), “TRJ” (*n* = 996)	Rice genotypic ensemble
AP.Variety*^§^	Categorical	Random	(334 levels)	Rice variety

Predictors used in shoot dry weight model are denoted by * and those in the hyphal colonization model are denoted with §.

### A Pot Experiment on 16 Cultivars

Using 16 cultivars selected to have a range of colonization in the screen of the RDP1, colonization rate was assessed in plants grown in 500 ml pots as described in the optimization steps above using a complete randomized block design with four replicate blocks. They were inoculated with 2% AM fungus inoculum, sown on the August 6th, 2015 and grown in the greenhouse as described earlier. After 32 days they were harvested and colonization assessed as described above.

### Genome Wide Association Mapping

Genome wide association mapping was performed with the 5.2 m SNP database (Wang et al., 2018) using a mixed model approach on all the cultivars implemented using EMMA (Efficient Mixed Model Analysis) (Yu et al., 2006), as described in Norton et al. (2014) (a SNP was selected as worth reporting if the *P*-value was <0.0001 and if the minor allele frequency (MAF) was >5%). The false discovery rate (FDR) of detected associations was estimated using the R-language Bioconductor “multtest” library to calculate Benjamini–Hochberg adjusted probabilities (Benjamini and Hochberg, 1995). A significance threshold of 10% FDR is normally used to identify putative SNP associations (McCouch et al., 2016) but it is not uncommon for no SNPs to reach this conservative threshold.

Significant SNPs within 200 kb of each other (based on consideration of linkage disequilibrium decay reported in this population as suggested by Zhao et al., 2011) were considered to represent SNP clusters of the same QTL and singleton SNPs were not considered QTLs (there must be at least 1 other SNP with *P* < 0.001 within 200 kb). Gene annotation was examined 200 kb either side of the most significant SNP of a QTL.

## Results

### Shoot Dry Weight

**FIGURE 3:**
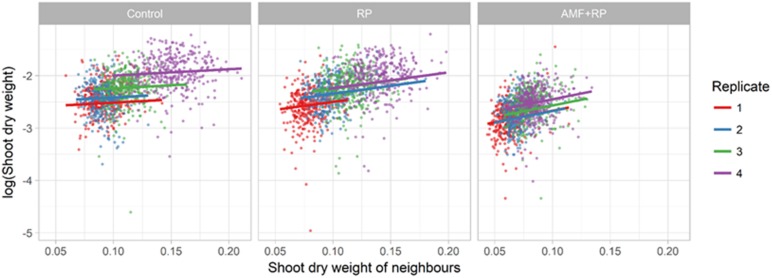
Plot of log shoot dry weight (SDW) of focal plant against the SDW of its neighbors, for each treatment. The positive effect is significant in all treatments (*P* < 0.005), with a slope increasing from Control to RP + AMF. For RP + AMF slope (effect) = 4.40 ± 0.53 (SE); for RP slope = 3.03 ± 0.39; for Control slope = 1.21 ± 0.40. Comparison of slopes between treatments are different as follows; Control to RP *P* = 0.0006; RP to RP + AMF *P* = 0.033.

The mean values for SDW and AM colonization rate are provided in Supplementary Table S1. SDW was affected by treatment such that the RP + AM plants were smaller while the other two treatments were only marginally different. Replicate runs, neighbor’s size, position within the box and cultivar all had an effect on SDW at least as large as treatment. With replicate run,

there was a gradual increase in SDW with number such that the averages were 78, 87, 102, and 125 mg per plant for runs 1–4, respectively. The spatial statistical analysis showed that plants on an external edge were slightly bigger than those that were not (103 mg vs. 98). More strikingly, plants on an internal edge were bigger than those that were not (110 mg vs. 97). The focal plant was larger when its neighbors were larger, and this positive effect of the neighbors’ average size was greater in the AM + RP than in the RP treatments, and least in the control treatment (Figure 3). The most parsimonious description of the spatial trends in plant SDW involved a significant spatial trend, common across all replicates (*P* = 1.28 × 10^–12^, Bayesian Information Criterion difference with models involving different spatial trends per box or block was greater than 15), which suggested an effect of the general orientation of the experimental setup in the greenhouse (see spatial trend in Supplementary Figure S1), independent of the position of the treatment in the box.

Once variation due to position and neighbors was removed from the data by subtracting their estimated effect from the observations, a two way analysis of variance (factors; treatment and genotype) on the SDW of the controls and rock phosphate treatment demonstrated a weakly significant effect of the rock phosphate treatment (*P* = 0.045) explaining less than 1% of the variation with rock phosphate stimulating growth (84.6 vs. 83.6 mg) and no cultivar by treatment interaction, while cultivar explained 68% of the non-spatial variation. This contrasts to ANOVA performed before the spatial analysis was conducted in which treatment and cultivar together only explained 30% of the total variation. When comparing rock phosphate to rock phosphate and AM fungus, the proportion of variation explained by treatment, cultivar and their interaction was 67% where it was only 49% before spatial correction. In this comparison, cultivar explained 50.1% of the variation, treatment 15.8% and the interaction 3.9%, the latter being small but highly significant (*P* < 0.001). Thus there is no evidence that cultivar relative performance differed between the control and rock phosphate treatment but it did when AM fungus was introduced. Ranking cultivars by their responsiveness to AM treatment (SDW ratio RP + AM:RP treatment) did not reveal any discernible pattern (between subpopulations or country/region of origin for example).

**FIGURE 4:**
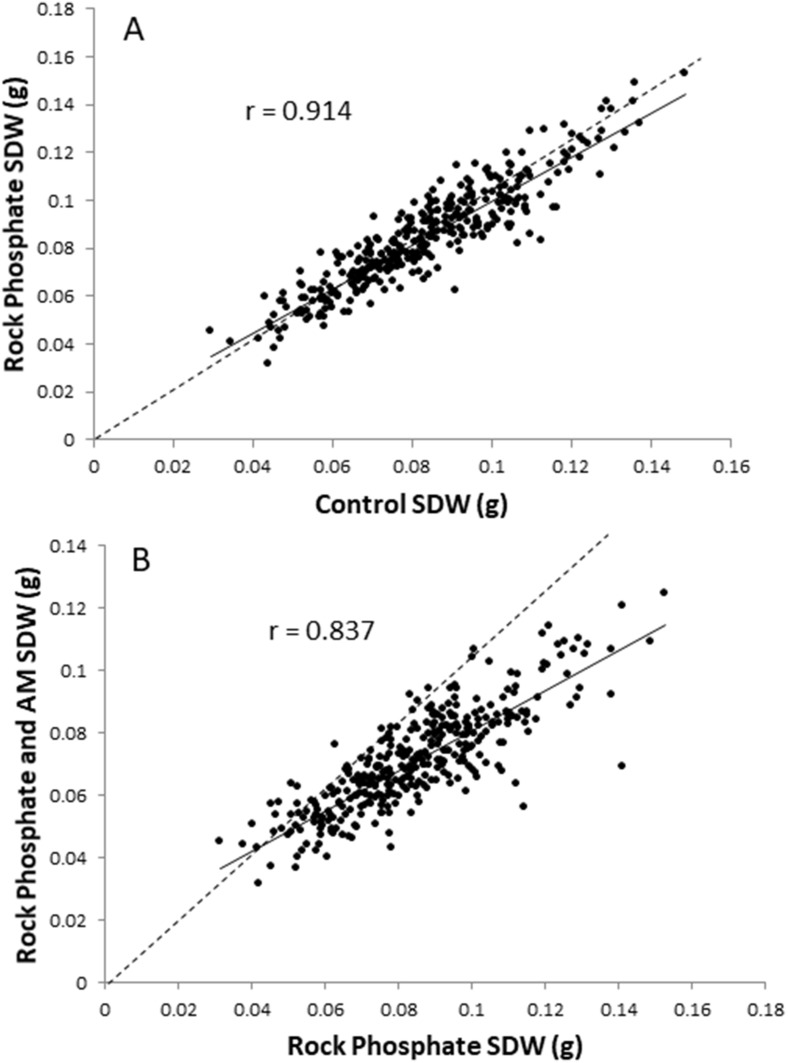
Plots of RDP1 cultivar mean SDW using spatially corrected data; **(A)** control vs. rock phosphate treatment; **(B)** rock phosphate vs. rock phosphate + AM fungus treatment. Solid line is regression, dotted line is 1:1 line.

Scatter plots of SDW for control vs. rock phosphate and for rock phosphate vs. rock phosphate plus AM fungus are shown in Figures 4A,B, respectively. These show the regression line and the 1:1 line (dashed). It clearly demonstrates the lack of effect of rock phosphate (Figure 4A), and the negative effect of AM fungus on SDW (Figure 4B). The correlation is better for the upper graph indicating that cultivar performance is more divergent in the comparison between rock phosphate and rock phosphate plus AM fungus.

**FIGURE 5:**
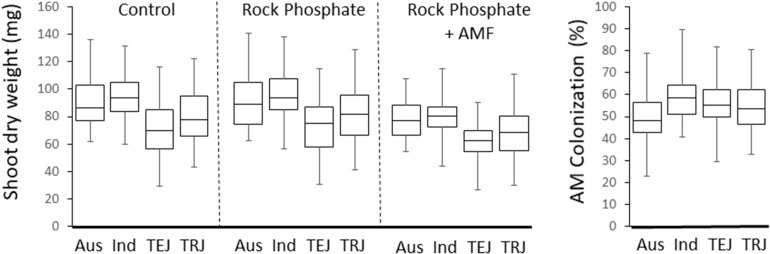
Boxplots of SDWs of each treatment, and hyphal colonization in the four main rice subgroups.

There was a difference in the SDW between subpopulations of the Rice Diversity Panel 1 which was consistent between treatments (*P* < 0.001, *R*^2^ = 14.7–18.2%), where aus (*n* = 54) and indica (*n* = 63) were high, tropical japonica (*n* = 83) were lower and temperate japonica (*n* = 82) were lowest (Figure 5).

### Hyphal Colonization

Hyphal colonization was roughly normally distributed in each run (Supplementary Figure S1) while both proportion of roots with arbuscles and vesicles were skewed toward low numbers and many zeros (Supplementary Figure S1). One way ANOVA on the raw data indicated heritability for proportion of roots with hyphae, arbuscles, and vesicles were 39, 29, and 35%, respectively. Mean hyphae colonization was highly correlated with mean arbuscles (*r* = 0.554, Supplementary Figure S1), and mean vesicles (*r* = 0.700, Supplementary Figure S2C). Based on these observations, it was decided that further analysis would be conducted on the hyphal colonization data alone.

**FIGURE 6:**
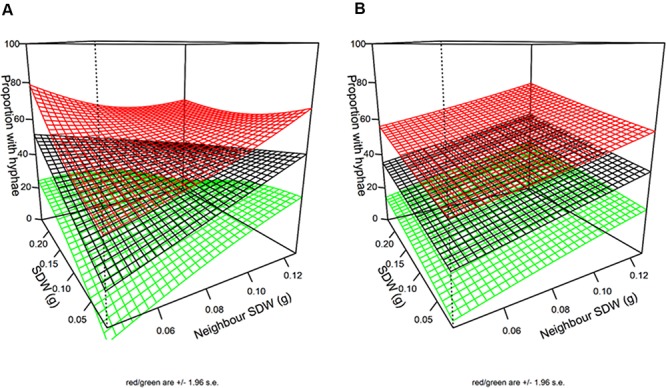
3D plots of the impact of neighbor and first order neighbor SDW (SDW) on hyphal colonization. The additive model **(A)** and the interactive model **(B)** are equally well supported by the data (AIC difference < 2). The black surface is the fitted model while the green and red surfaces are the 95% confidence intervals. Units are g for SWD and % colonization for hyphae.

Spatial analysis indicated hyphal colonization of a particular plant increased with its SDW (*P* = 0.026) and the mean SDW of its nearest (8) neighbors (*P* = 0.0012). The interaction between the effects of focal plant and neighbors’ SDWs was close to significant (*P* = 0.066) (Figure 6B) so the data did not provide clear support for one model over the other. In other words, the difference in the predictions of the simple additive model and of the interactive model was small (Figures 6A,B). Both models agreed that large SDW of either the focal plant or its neighbors increased hyphal colonization of focal plant in similar ways.

**FIGURE 7:**
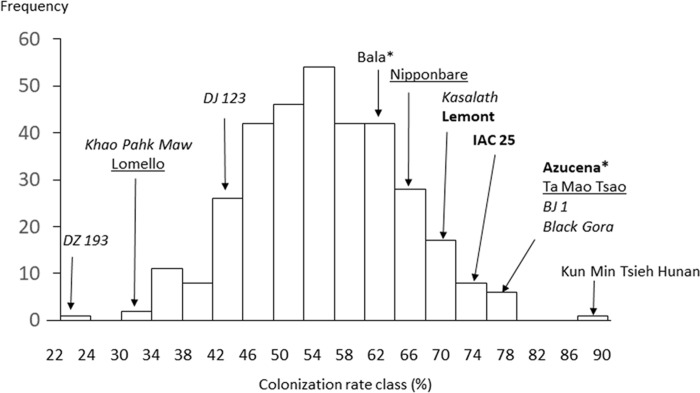
Frequency distribution of mean hyphal colonization in the Rice Diversity Panel 1 cultivars highlighting values of some notable cultivars. * for Azucena and Bala indicates these are the Aberdeen “own” genotypes for these cultivars. The font used for cultivars indicate subpopulation; normal = indica, underlined = temperate japonica, italic = aus, bold = tropical japonica.

The model indicated significant and idiosyncratic spatial trends in each of the four replicates (Markov Random Field component: *P* < 2 × 10^–16^; see Supplementary Figure S3). This spatial analysis allowed for a correction of estimation of the colonization of the plants such that the proportion of variation in colonization explained by cultivar (from one way ANOVA) increased from 35 to 42% with heritability increased from 39 to 54%. The mean colonization rates for each cultivar are presented in Supplementary Table S1 while Figure 7 shows a histogram of the distribution. The average coefficient of variation for the trait was 0.25 (it was 0.30 before spatial analysis). The range in colonization was considerable, from the Bangladesh aus cultivar DZ 193 at 22.9 ± 8.3% (±standard deviation) to the Chinese indica cultivar Kun Min Tsieh Hunan at 89.8 ± 9.3%. Colonization rates differed between subgroup (*P* < 0.001) where indica, temperate japonica and tropical japonica were similar (58.0, 54.5, and 55.0%, respectively) while aus were lower (50.0%) (Figure 5). Within each subgroup there were large variations but it is considered noteworthy that while there were several aus cultivars in the top 30 cultivars, there were no indicas in the lowest 30 cultivars. Differences were also detected between countries or regions (country groups when individual countries had less than 10 cultivars) (*P* < 0.001, *R*^2^ = 12.7%) where the Philippines and China were high and Bangladesh and United States were low (Supplementary Figure S4). These differences are probably partly driven by different subgroup compositions for countries (e.g., Bangladesh is dominated by aus cultivars) but in the tropical japonica subgroup there is still a detectable country/region difference (*P* = 0.006, *R*^2^ = 26.3%) where the six Philippine cultivars had higher colonization (71.9%) than 13 South American (55.7%), 15 West African (55.0%), 10 SE Asian (55.0%), and 15 United States (49.5%) cultivars.

### Pot Experiment on 16 Cultivars

**FIGURE 8:**
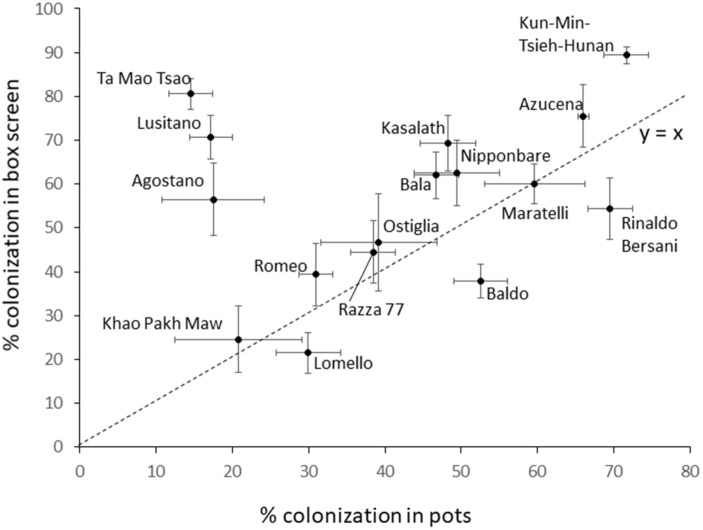
Mean hyphal colonization for 16 cultivars tested in both the large box screen, and individual pots. Bars are standard error. Dotted line is the 1:1 line.

The colonization of 16 cultivars grown in pots differed significantly (*P* = 0.002) and cultivar explained 50% of the variation. Conducting a two way ANOVA on colonization data from both the full RDP1 screen and the pot experiment with factors cultivar and method (pot or RDP screen) found the model explained 62% of the variation and indicated all factors were significant at *P* < 0.001, including the cultivar by method interaction (*F* = 27.8, 5.6, and 3.1 for cultivar, method and interaction, respectively). Colonization in pots is plotted against the results obtained in the RDP1 screen in Figure 8. There is no correlation between the colonization rates obtained in the two experiments. However, it is remarkable how close most of the cultivars are to the 1 to 1 line indicating most performed very similarly in both experiments. Three cultivars (Ta Mao Tsao, Lusitano and Agostano) appear to have very substantially different colonization rates in the pot. Indeed, if these three are removed, the remaining 13 cultivars correlate with *r* = 0.806.

### GWA Mapping

**FIGURE 9:**
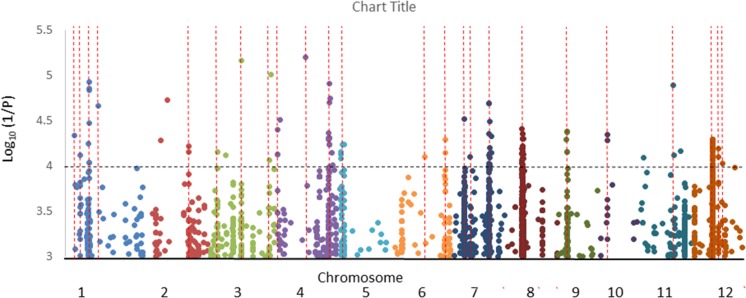
Genome wide association mapping of the All analysis showing only SNPs where *P* < 0.001. Lines indicate where there is at least one other marker within 200 kb. Each color represents a different chromosome.

The results of GWA mapping for the hyphal colonization with all subgroups is presented in Supplementary Figure S5 and summarized in Table 2 while the results of analysis with the All analysis (all cultivars) is graphed for more clarity in Figure 9. No SNPs were considered significant using the FDR of 10%. In total 23 putative QTLs were revealed in the analysis of all cultivars using the criteria that for at least 1 SNP *P* < 0.0001 and MAF > 5% and there is at least one other SNP with *P* < 0.001 within 200 kb. A further two putative QTLs were identified either in the aus subpopulation or both the aus and tropical subpopulation. No putative QTLs were detected in the indica or temperate japonica subpopulations. Genes within 200 kb of the most significant SNPs were listed and their responsiveness to AM colonization as reported by Fiorilli et al. (2015) and Gutjahr et al. (2015) is given in Supplementary Table S2. The number of genes in the lists ranges from 50 to 99 and they had an average of 8.8% of genes being differentially expressed in either Fiorilli et al. (2015) or Gutjahr et al. (2015) or both.

**TABLE 2:** The most significant SNP at each of the 23 identified GWA putative QTLs for hyphal colonization across all populations plus three from subpopulation analysis.

QTL	Analysis	Most significant SNP id	Chromosome	Position (bp)	*P*-value	MAF (%)	Effect
1.1	All	mlid0092747457	1	5069003	4.60E-05	7.3	4.6
1.2	All	mlid0001644797	1	8119524	7.58E-05	20	4.0
1.3	All	mlid0002847008	1	13328067	3.37E-05	21	-4.6
1.4	All	mlid0004161541	1	18083827	2.15E-05	39	-5.0
2.1a	Aus	mlid0014401085	2	22623115	7.49E-06	44	-12.7
2.2	All	mlid0014856649	2	24494645	6.07E-05	31	2.7
3.1	All	mlid0017818601	3	4455505	7.02E-05	31	-5.1
3.2	All	mlid0020494652	3	17569164	6.83E-06	45	5.4
3.3	All	mlid0023902122	3	32806262	8.53E-05	11	-7.0
4.1	All	mlid0024944825	4	1252233	3.92E-05	14	8.6
4.2	All	mlid0029088874	4	16530764	6.25E-06	10	5.3
4.3	All	mlid0031764657	4	29353046	2.87E-06	42	3.7
5.1	All	mlid0033051374	5	774066	6.90E-05	19	-4.8
6.1	All	mlid0043758793	6	17006825	7.81E-05	11	6.2
6.2	All	mlid0046376100	6	28107199	5.11E-05	24	-4.8
7.1	All	mlid0048451088	7	7182668	3.04E-05	31	-4.8
7.2	All	mlid0052022846	7	20886439	4.41E-05	14	9.1
8.1	All	mlid0056191014	8	9715485	4.38E-05	22	3.6
9.1	All	mlid0062250420	9	5765734	4.16E-05	41	5.7
10.1	All	mlid0067485302	10	4753692	4.47E-05	7.3	-5.1
11.1	All	mlid0076234181	11	17686395	1.29E-05	20	-5.1
11.2tr	Trj	mlid0077311363	11	21793808	1.81E-05	12	-8.1
11.2a	Aus	mlid0094185850	11	22027960	6.21E-05	10	-10.8
12.1	All	mlid0081838150	12	10779534	5.12E-05	19	4.3
12.2	All	mlid0082869441	12	14246478	6.38E-05	23	5.2
12.3	All	mlid0083460931	12	16261109	9.29E-05	38	4.5

## Discussion

### Cultivars Differ in Colonization by *R. irregularis* and the Impact of the AM Fungus on Plant Growth

The main aim of this study was to test the hypothesis that there is genetic variation within rice for colonization and the impact of the AM fungus on rice growth. The study clearly showed there is. Firstly, there was highly significant differences between cultivars in colonization with a very wide range from 23–90% (Figure 7). Secondly, it shows that impact of AM fungus on shoot growth, all be it almost entirely negative, is different between cultivars (Figure 4B). There are few studies showing cultivar differences in rice. A meta-analysis of the impact of AM fungi on annual crop plants has been conducted on 39 papers containing 320 species by Lehmann et al. (2012) which concluded that cultivars differed in colonization rates, and interestingly old and new cultivars had less colonization that ancestral ones, and that colonization rate was quite strongly correlated to responsiveness (of host growth) to AM fungi. However, the analysis included only one study specific to rice (Gao et al., 2007). Gao et al. (2007) pot experiments found one of six aerobic rice cultivars had lower root colonization. Differences between an upland and a lowland cultivar were found to depend on the species of AM fungus used and the amount of arsenic applied as a treatment (Li et al., 2011). Suzuki et al. (2015) assess 64 rice cultivars for growth response to a different AM fungus and measured colonization in 12 of them. Only two cultivars are in common between that study and the current one (Nipponbare and Kasalath) and while they appeared to differ in growth response in Suzuki et al. (2015), here they do not. Considering examples of studies within other grass species, differences in five Canadian durum wheats have been reported including an interaction between soil fertility and cultivar (Singh et al., 2012). Amongst six winter wheats and maize accessions, Zhu et al. (2001) found cultivar differences, while Chu et al. (2013) found differences in four maize cultivars but not at the lowest P addition. Recently three studies have examined a wide range of either sorghum (Leiser et al., 2016), or wheat (Lehnert et al., 2017) or durum wheat (De Vita et al., 2018). Leiser et al. (2016) tested 187 sorghum cultivars grown in pots 38 days and found only limited variation in AM fungal colonization rates in terms of range (38.5–72.9%) or heritability (15%). Lehnert et al. (2017) tested 94 wheat cultivars grown in pots until mature. They showed very strong genotypic differences but a lower range of colonization (24–56%). De Vita et al. (2018) found large differences in colonization of durum wheat roots with both AM fungi *R. irregularis* and *F. mosseae*, and discovered seven QTLs apparently common between the species suggestive of common genetic regulation within the host.

The differential cultivar response of shoot growth to AM fungal plus rock phosphate treatment relative to the rock phosphate treatment alone detected in this experiment was highly significant but small and did not fall into any discernible pattern (e.g., between subgroups or country of origin). This implies the host genetics of rice-AMF interaction are influential in determining plant growth, but much more research is needed to determine its biological significance. Despite the high statistical difference and big range of variation in colonization detected here, there is no particularly striking pattern that provides conclusive evidence of the biological implications. While aus cultivars are generally lower than other subpopulations (Figure 5), and there are differences related to the country of origin of the cultivars, these do not explain a large proportions of the variation or provide clear reasons for such differences. Aus cultivars, which are revealed to have a low colonization on average, are notable as the donors of a number of important abiotic resistance traits [e.g., submergence tolerance from FR 13A (Xu et al., 2006); phosphorus starvation tolerance from Kasalath (Gamuyao et al., 2012); drought resistance from N22 and Dular (Gowda et al., 2011)] and might be expected to be adapted to poor soils and might therefore be expected to have strong associations with AM fungi. It is perhaps notable then that Kasalath does have a high colonization rate (ranked 19th) while the other three cultivars mentioned above are not remarkable. Kasalath was identified as having high phosphorus uptake efficiency of 30 rice genotypes in a study which also identified the Brazilian tropical japonica IAC 25 as high for P uptake efficiency and the United States tropical japonica Lemont as low (Wissuwa and Ae, 2001). IAC 25 is ranked 9th here so is high for AM colonization, but Lemont ranked 33rd for colonization which is definitely not low.

The fact that cultivars from the Philippines and China have high root colonization and those from Bangladesh are low (Supplementary Figure S2) is not currently explicable. One hypothesis that could be forwarded would be that upland cultivars adapted to aerobic soils would have a greater need of, and therefore stronger association with AM fungi than flooded cultivars. Unfortunately information on the hydrological environment of the cultivars in the RDP1 is lacking.

The repeated test of 16 cultivars in a pot experiment produced an interesting result (Figure 9), being a remarkably strong agreement in colonization rates between the large box screen and pots for 13 of the cultivars, but for three cultivars their colonization in pots was much lower than in the box screen. It can be hypothesized that this reflects cultivar differences in the impact of soil properties on colonization. The preliminary experiments reported here demonstrate that colonization of Bala was sensitive to rock phosphate while in Azucena it was not. Since Bala was included amongst the 16 tested in pots, and its colonization was very similar to the box screen colonization rate, it seems unlikely the same edaphic factor is acting in the pot experiment reported in Figure 8 and the preliminary experiments. The cultivar difference in the impact of edaphic factors on root colonization by AM fungi merits further study.

### Impact of *R. irregularis* on the Rice Plants

The optimization experiments do not provide convincing evidence that P uptake efficiency and root colonization by AM fungi are linked, which may occur because P uptake is more likely to be related to proliferation of extra-radical mycelium (Sawers et al., 2017), or that under these conditions, *R. irregularis* is behaving toward the parasitic end of the plant-mycorrhiza continuum (Johnson, 2010). It will be important to test if different root colonization rates detected here reflect colonization obtained with other species of AM fungi and colonization in the field environment.

In the preliminary experiments and the main screen described in this report, the AM treatment invariably reduced plant growth, which is not a unique observation (Van Der Heijden and Horton, 2009). We would suggest that this is a feature of the soil medium used here since other experiments in Aberdeen with the same rice genotypes and the same AM fungal inoculum, but with different growing medium, do not always have a negative effect. What is highly remarkable from study is the strong positive neighbor effects detected on both plant growth and root colonization in the presence of *R. irregularis*. The positive nature of these effects of neighbors suggest *R. irregularis* has formed a common mycorrhizal network (CMN) inter-connecting roots of individual plants (Selosse et al., 2006). Recent work has shown that CMNs can affect growth and nutrition of neighbors grown in pairs (Walder et al., 2012). This should be tested on rice to determine the repeatability, extent and mechanism of this apparent facilitation. If proven more than just specific to the conductions used in this experiment, the result has major implications for consideration of mycorrhizal crop plants and their productivity since it demonstrates that when connected by a CMN, one plant can have a positive impact on its neighbors. This phenomenon merits further study, especially at the field level.

### Putative QTLs for Root Colonization by AM Fungi by GWA Mapping

In this study, 23 putative QTLs for root colonization by AMF were detected, mostly in the analysis of the whole population. Two important observations need to be made. First, no association was above the 10% FDR; this does not mean they are not real, but they must be treated with caution, hence they are termed putative QTLs. Second, the number is high. If these QTLs are to be believed, the implication is that there are many genes involved in determining the degree of colonization in rice–AM fungal symbiosis and the trait is truly quantitative. In the study of sorghum (Leiser et al., 2016) the GWA mapping revealed no SNP associated with colonization with a *P*-value below *P* = 0.0001 which they considered reflected the low heritability (15%). In contrast, the wheat study of Lehnert et al. (2017), which had a heritability of 54% for root colonization (the same as reported here for rice), found six QTLs. More studies are needed before we can determine if root colonization in grasses is generally regulated by multiple, small effect genes as implied in the studies to date.

It is worth noting that none of the QTLs reported here match the 97 and 16 loci found associated with either controlled environment or field resistance to the fungal pathogen blast in this population [as reported by Kang et al. (2016) and Zhu et al. (2016), respectively]. This implies that the loci reported for AM colonization are not associated with genes affecting the basic plant/fungus interaction process.

### Candidate Genes for Putative QTLs

Identifying the most promising functional candidate genes from the gene lists for the 23 putative QTLs reported here (Table 2 and Supplementary Table S2) will depend on individual and community understanding of the molecular mechanisms involved in the AM fungus-rice interaction which will improve with time. However, some notable observations on examination of these lists are given below.

In QTL 1.1 is LOC_Os01g09580, a calcium/calmodulin serine/threonine protein kinase which is massively downregulated in AMF in both the Fiorilli et al. (2015) and Gutjahr et al. (2015) study. Over expression in rice of *Medicago* DMI3 (a Ca+/calmodulin-dependent serine/threonine protein kinase that is known to be a component of the common symbiotic pathway between AM fungi and *rhizobium*) supported elevated AM fungal colonization (Ortiz-Berrocal et al., 2017).

In QTL 2.2 is a pair of excellent candidate genes. The genes LOC_Os02g40710 and LOC_Os02g40730 are annotated as ammonium transporters. These genes are *OsAMT1.2* and *OsAMT1.3*, respectively. The transport of ammonia from AM fungi to plant host within the arbuscule is considered a central process in AM symbiosis (Guether et al., 2009). Transcriptomics studies on rice exposed to *R. irregularis* identified ammonium transporters (although not these two) as responsive (Güimil et al., 2005; Gutjahr et al., 2015), while the expression of ammonium transporter *OsAMT3.1* has been used as a marker for rice-AM interaction (Vallino et al., 2014). Furthermore, AMT2:3 appears to be important in influencing the life span of *Medicago*-associated AM fungi (Breuillin-Sessoms et al., 2015). Fiorilli et al. (2015) revealed that LOC_Os02g40730 is strongly upregulated by *R. irregularis* inoculation (2.2-fold in large laterals, 9-fold in fine laterals). Both *OsAMT1.2* and *OsAMT1.3* have a highly root-specific expression pattern in rice (Sato et al., 2013).

In QTL 4.1 is a cluster of seven subtilisin or subtilisin-like proteases including LOC_Os04g02980 annotated as OsSub33 which is upregulated in both root types in the Fiorilli et al. (2015) study. Subtilisins are a class of serine proteases also known as subtilases. A review on subtilases (Schaller et al., 2017) highlights studies that suggests some subtilases are linked to AM colonization, having a role in arbuscule development. Further, a subtilase was identified by Kistner et al. (2005) as one of seven host genes required for mycorrhiza and symbiotic bacteria to enter root epidermal or cortical cells based on the study of a mutant of *Lotus japonicus* that are impaired for nodulation. While this makes these genes good candidates for rice-AM fungal interaction, it must be noted that there are 71 genes in rice annotated as subtilisin-like or Subtilisin homologs.

Recently a 53 kbp deletion at 29 Mbp on chromosome 1 regulating strigolactone production and strongly associated with resistance to the parasitic plant *Striga* was reported (Cardoso et al., 2014). This deletion was tested by PCR on the RDP1 and used as a marker in the GWA mapping conducted here. It is surprising perhaps that there is no association detected at this locus since the production of strigolactone is considered to be an evolutionary adaptation critical to the signaling by plants to mycorrhiza (Akiyama et al., 2005).

## Conclusion

These experiments clearly demonstrate that rice cultivars differ very widely for colonization by *R. irregularis* and reports putative QTLs which appear to contribute to that variation. While the pattern of variation in colonization in rice offers little insight into the evolution or biological significance, it lays a foundation for further research. This may be determining if variation detected here matches variation in the field and if it impacts nutrient uptake in low nutrient, aerobic environments. Some of the genes under the QTLs shown here should be further investigated. A supplementary finding is strong evidence of positive interactions between neighboring rice plants facilitated by a common mycorrhizal network.

## Author Contributions

AP conceived and designed the experiments and largely wrote the manuscript. HD conducted preliminary experiments and the screen of RDP1. RS conducted the study of 16 cultivars in pots. TC did the spatial statistical analysis. AD and TT conducted the GWA mapping. DJ advised on the role of mycorrhiza in common mycorrhizal networks. All authors contributed to editing the manuscript.

## Conflict of Interest Statement

The authors declare that the research was conducted in the absence of any commercial or financial relationships that could be construed as a potential conflict of interest.

## Supplementary Material

FIGURE S1 Spatial trend in shoot dry weight (SDW), shared between all four replicate runs (showing deviations from predicted value, on the log-scale in grams). Values increase from red to white.

FIGURE S2 Raw AM colonization data showing; **(A)** frequency distribution of hyphal colonization in each run; **(B)** scatter plot of mean hyphal colonization vs. mean arbuscle colonization and; **(C)** hyphal colonization vs. mean vesicle colonization.

FIGURE S3 Spatial trends in hyphal colonization in each of the four replicate runs (deviation from predicted value). Units are % colonization.

FIGURE S4 Boxplots of hyphal colonization in Rice Diversity Panel 1 according to country of origin (with *n* in brackets). Letters above bars represent results of Tukey’s test of difference between groups.

FIGURE S5 Manhattan and QQ plots for hyphal colonization for all cultivars, aus (AUS), indica (IND), tropical japonica (TRJ), and temperate japonica (TEJ) cultivars. Red dotted lines indicate putative QTLs from the all analysis, green in subgroups.

TABLE S1 List of rice accession and phenotype data.

TABLE S2 List of genes observed in each of the putative QTLs with information on available transcriptomics data for response to AM treatment [using data from Fiorilli et al. (2015) or Gutjahr et al. (2015)].
