# Supplementary figures and images for: Spatial Effects and GWA Mapping of Root Colonization Assessed in the Interaction Between the Rice Diversity Panel 1 and an Arbuscular Mycorrhizal Fungus
Source: Front Plant Sci. 2019 May 17;10:633. doi: 10.3389/fpls.2019.00633 (PMC6533530; doi:10.3389/fpls.2019.00633)

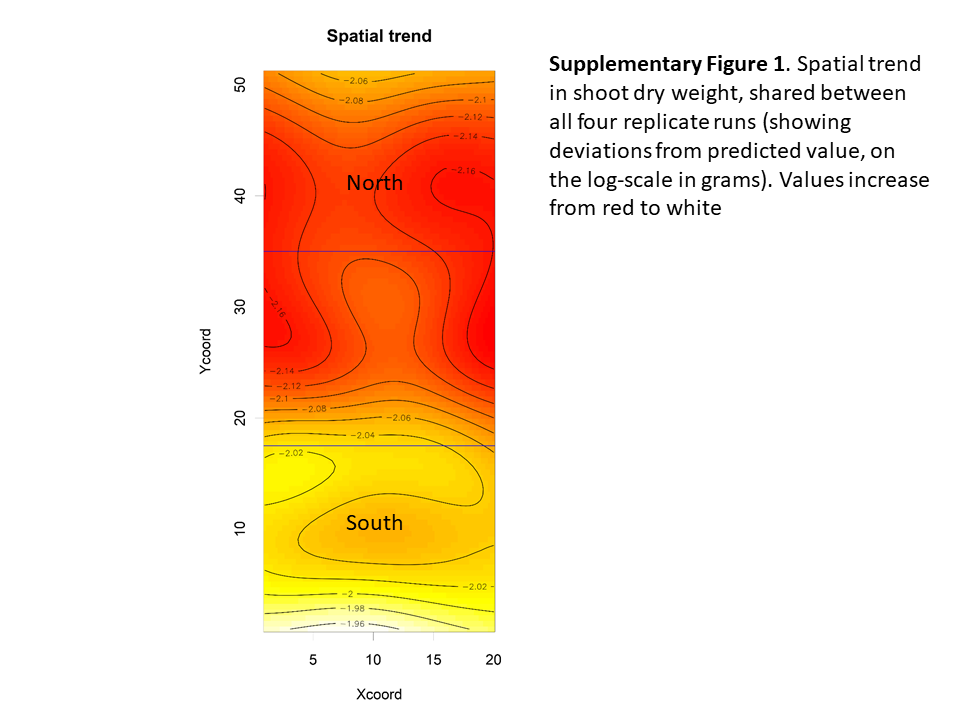

Supplement: FIGURE S1 — Spatial trend in shoot dry weight (SDW), shared between all four replicate runs (showing deviations from predicted value, on the log-scale in grams). Values increase from red to white. [file Image_1.TIF]

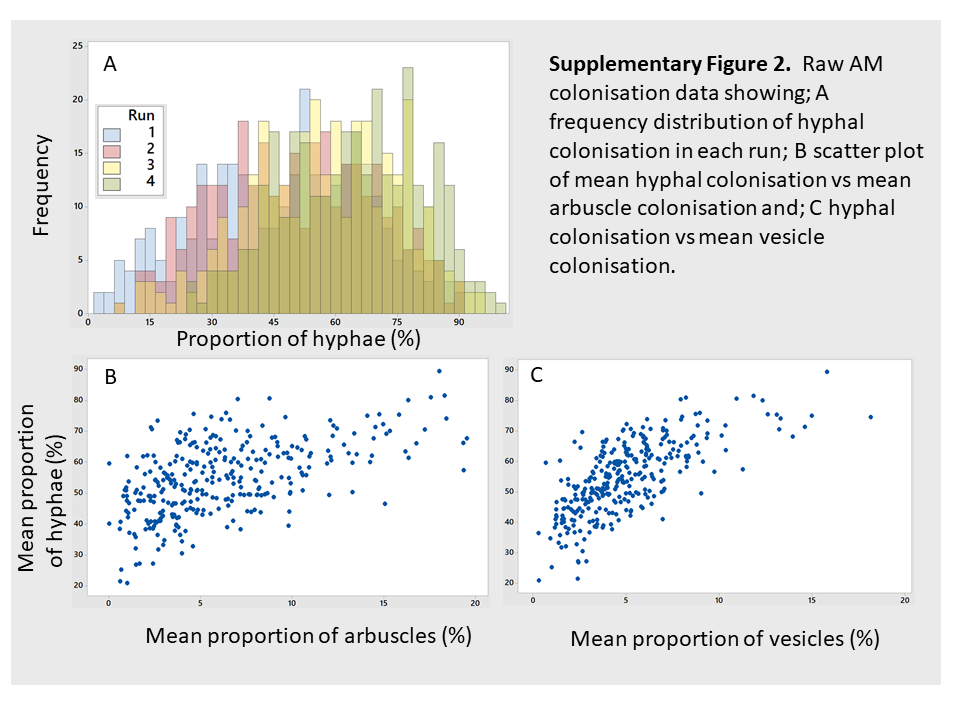

Supplement: FIGURE S2 — Raw AM colonization data showing; (A) frequency distribution of hyphal colonization in each run; (B) scatter plot of mean hyphal colonization vs. mean arbuscle colonization and; (C) hyphal colonization vs. mean vesicle colonization. [file Image_2.tif]

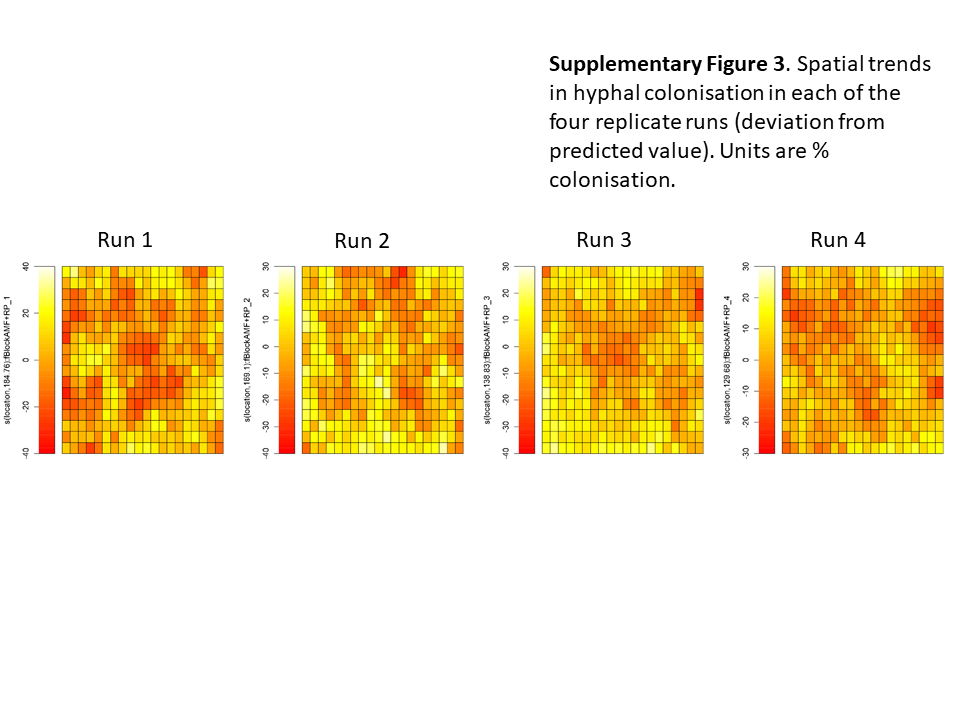

Supplement: FIGURE S3 — Spatial trends in hyphal colonization in each of the four replicate runs (deviation from predicted value). Units are % colonization. [file Image_3.TIF]

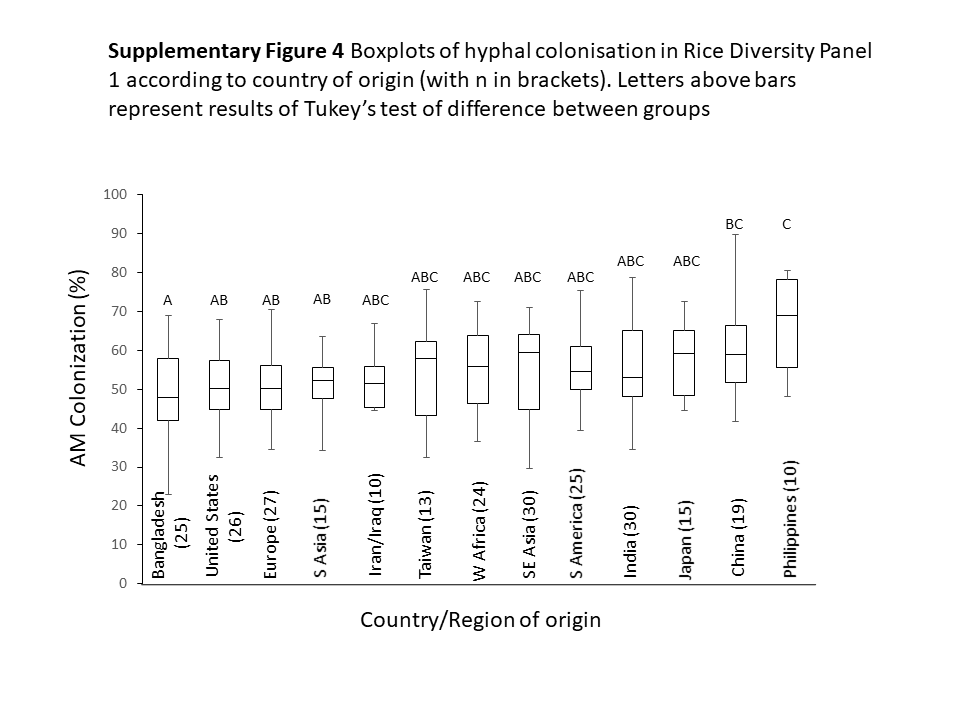

Supplement: FIGURE S4 — Boxplots of hyphal colonization in Rice Diversity Panel 1 according to country of origin (with n in brackets). Letters above bars represent results of Tukey’s test of difference between groups. [file Image_4.TIF]

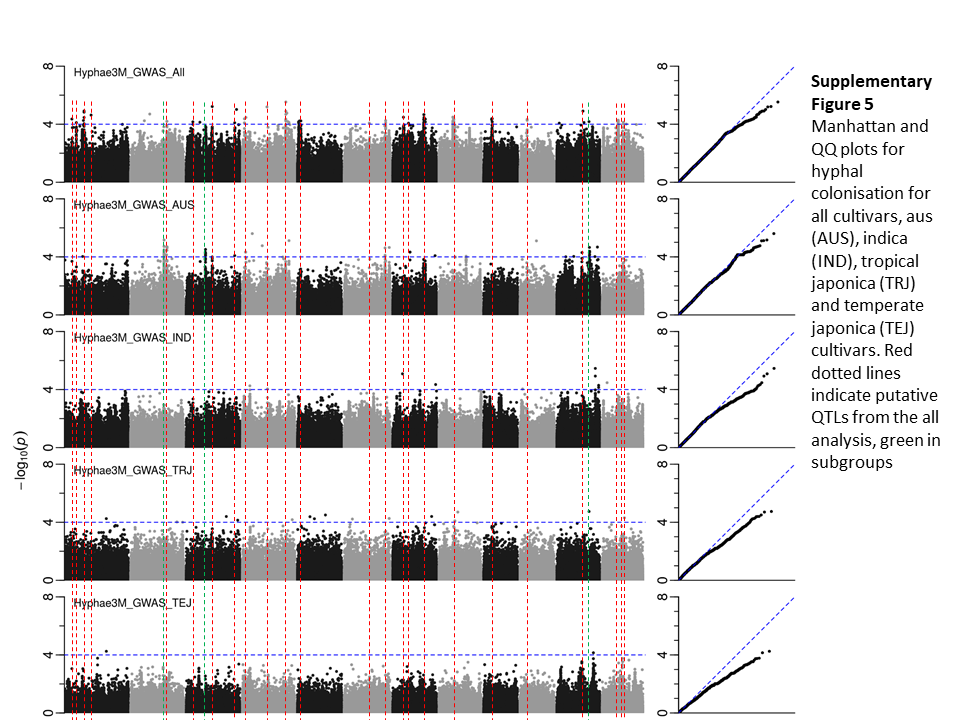

Supplement: FIGURE S5 — Manhattan and QQ plots for hyphal colonization for all cultivars, aus (AUS), indica (IND), tropical japonica (TRJ), and temperate japonica (TEJ) cultivars. Red dotted lines indicate putative QTLs from the all analysis, green in subgroups. [file Image_5.TIF]
